# Single step track dilatation for percutaneous nephrolithotomy in children

**DOI:** 10.1007/s11255-022-03314-1

**Published:** 2022-08-08

**Authors:** Ahmed Fahmy, Wally Mahfouz, Mohamed Elbadry, Ahmed Moussa

**Affiliations:** 1 Urology Department, Alexandria University, Alexandria, Egypt; 2 Urology Department, Minia University, Minya, Egypt

**Keywords:** Dilatation, Single step, Sequential, Percutaneous nephrolithotomy, Children

## Abstract

**Introduction and objectives:**

Data on the use of single step dilatation technique during pediatric percutaneous nephrolithotomy (PCNL) in the literature is sparse. In this prospective randomized study, we aimed to compare the safety, efficacy, and perioperative complications of single step versus serial tract dilatation using Alken metal telescopic dilators during pediatric PCNL.

**Methods:**

Patients undergoing PCNL were randomized into two groups according to the dilatation technique used. In group A, Alken telescopic serial metal dilatation was utilized, and in group B, single step dilatation was performed. Inclusion criteria included children < 18 years with stone burden from 2 to 4 cm, located in the renal pelvis ± one calyx, who were candidates for PCNL.

The primary outcomes were access time and complications’ rate. The secondary outcomes were dilatation fluoroscopy time, operative duration, stone free rate, postoperative hospital stay, hemoglobin deficit, and need for blood transfusion. Both outcomes were evaluated and compared between both treatment groups.

**Results:**

A total of 70 patients were randomized into group A (35 patients) and group B (35 patients). Access was successfully obtained in all procedures. All the procedures were performed through a single tract.

Access time and dilatation fluoroscopy time were shorter in group B (statistically significant). Patients in group A had higher rate of complications (statistically significant). Intraoperative bleeding requiring blood transfusion was less in single track dilatation than serial metal track dilatation.

**Conclusions:**

Compared to serial metal track dilatation, single step dilatation showed comparable operative time and stone free rate, with significantly reduced access time and dilatation fluoroscopy time.

## Introduction

Percutaneous nephrolithotomy (PCNL) is an established treatment modality for large, multiple, and complex kidney stones in children [1]. However, it may present a peculiar intraoperative challenge because of the high mobility, small size of the kidney in children and its need for high endoscopic expertise [2].

Establishing a safe and effective percutaneous tract during PCNL procedure is a fundamental step for procedure success. Since the introduction of PCNL, several techniques have been proposed to make tract establishment and dilatation easier, safer and with less complications. Complications such as dilatation failure, collecting system perforation, urinary extravasation, intraoperative bleeding, and injury of adjacent structures are usually directly related to the tract dilatation procedure [3].

Percutaneous tract dilatation is performed mainly by four methods: metal telescopic Alken dilation, Amplatz serial dilatation, balloon dilatation and one-stage dilatation method. The one-stage technique was introduced by Frattini et al. [4]. In this procedure, dilatation is performed using 25- or 30-F Amplatz dilators over the guidewire to dilate the tract in a single step.

Single step dilatation technique has been reported to be safe, effective, and accompanied with shorter duration of surgery, and less radiation exposure time in many studies [5, 6]. In contrast, other reports have shown that single step dilatation may cause more trauma to the renal parenchyma than sequential dilatation [7].

Data on the use of this technique during pediatric PCNL in the literature is sparse. In this study, we aimed to compare the safety, efficacy, and perioperative complications of single step versus serial tract dilatation using Alken metal telescopic dilators during pediatric PCNL.

## Materials and methods

Between April 2017 and May 2021, seventy children who were candidates for PCNL were enrolled in this prospective randomized study. Inclusion criteria included children aged < 18 years who had kidney stones and were candidate for PCNL without contraindications. Stone burden was from 2 to 4 cm, located in the renal pelvis ± one calyx. Exclusion criteria were any contraindications for PCNL, congenital renal anomalies and previous renal surgery.

This study was approved by the Institutional Review Board at our institution. The endoscopic intervention was explained to parents or patients’ guardian, and written informed consent was obtained.

### Preoperative evaluation and randomization

All patients were assessed preoperatively by ultrasonography and non-contrast spiral computed tomography. Routine blood tests and urine cultures were obtained. Patients with urinary tract infection received antibiotics according to sensitivity tests.

Eligible patients were randomized using sealed opaque envelopes into two treatment groups according to the dilatation technique used. In group A. Alken metal telescopic dilatation was utilized to dilate the percutaneous tract, and in group B, single step dilatation technique was performed.

### Intervention

All procedures were done with the patient under general anesthesia in the standard prone position. Antibiotic prophylaxis with a first-generation cephalosporin (cephazolin) was routinely administered to all patients. Cystoscopy was performed with retrograde insertion of a 5 Fr open ureteric catheter, followed by calyceal puncture using an 18-gauge angiographic needle (two-part trocar needle, M/S Cook Medical), under fluoroscopic guidance with insertion of both working and safety guidewires. The percutaneous tract was initially dilated by insertion of an 8-F polyurethane dilator.

In group A, the tract was dilated up to 20-F using sequential Alken metal dilators. In group B, a single 20-F Amplatz dilator (M/S Cook Medical Amplatz Renal Dilators) was passed over the Alken guide wire. The surgeon then applied twisting movements with slowly advancing the dilator into the collecting system, followed by advancing the access sheath over the dilator under fluoroscopic guidance. In both groups, a 20-F Amplatz sheath was inserted after tract dilatation. A 17-F semirigid nephroscope was used and a pneumatic lithotripter (Litho Crack, Sp. Swiss-Germany) was used for stone disintegration. Warm normal saline was used for irrigation. Fluoroscopy was used for confirmation of stone clearance after completion of stone fragmentation and retrieval. A 14-F nephrostomy tube was placed at the end of the operation. All cases were performed by a single surgeon who had an experience in pediatric PCNL.

### Postoperative evaluation and follow-up

Hemodynamic parameters, hemoglobin concentration, hematocrit level and intraoperative and postoperative need for blood transfusion were evaluated and recorded during the first 24 h postoperatively.

Plain X-ray abdomen and pelvis and ultrasonography were performed postoperatively, before removal of the nephrostomy tube, for detection of any residual fragments. This was performed for all patients after one month, for confirmation of stone-free status. Perioperative complications were recorded and classified according to the modified Clavien–Dindo grading system [8]. The postoperative hospital stay was calculated for each group.

### Outcome measures

The primary outcomes were access time and complications’ rate. The secondary outcomes were dilatation fluoroscopy time, operative duration, stone free rate, postoperative hospital stay, hemoglobin deficit, and need for blood transfusion. Both outcomes were evaluated and compared between both treatment groups. The assessor conducting analyses of the outcomes was blinded to the treatment groups.

Access time is defined as the time interval from the initial renal fluoroscopic imaging to successful placement of the Amplatz sheath into the collecting system. Dilatation fluoroscopy time is defined as the number of seconds of X-ray exposure that elapsed from the time of placement of the guidewire into the collecting system until the insertion of the Amplatz sheath. The operative duration was calculated from the time of initial cystoscopic ureteral catheter placement until securing the 20-F nephrostomy tube. Stone-free status was defined as no residual fragments on KUB and ultrasound urinary tract after 1 month. The hemoglobin deficit was the difference between the preoperative level and its level 12 h postoperatively.

### Statistical analysis

We used the G*Power program (http://www.gpower.hhu.de/en.html) for sample size calculation. A sample size of 64 was calculated to detect a 10% difference in the complications rate used as a primary outcome between the two groups and to provide an 80% power for the study. Only patients who completed the 1 month follow-up underwent the final analysis.

Data collected were analyzed using a commercially available statistical program (SPSS version 20; IBM Corporation; Armonk, NY, USA). Continuous variables were tested for normal distribution using the Kolmogorov–Smirnov test, are presented as mean ± standard deviation (normal distribution) and median (range) (non-normal distribution) and were analyzed using the independent sample *t*-test or Mann–Whitney *U*-test. Categorical data are presented as numbers (%) and were analyzed using the chi-square test or the Fisher exact test, as appropriate. *P* < 0.05 considered to indicate statistical significance.

## Results

**Fig. 1:**
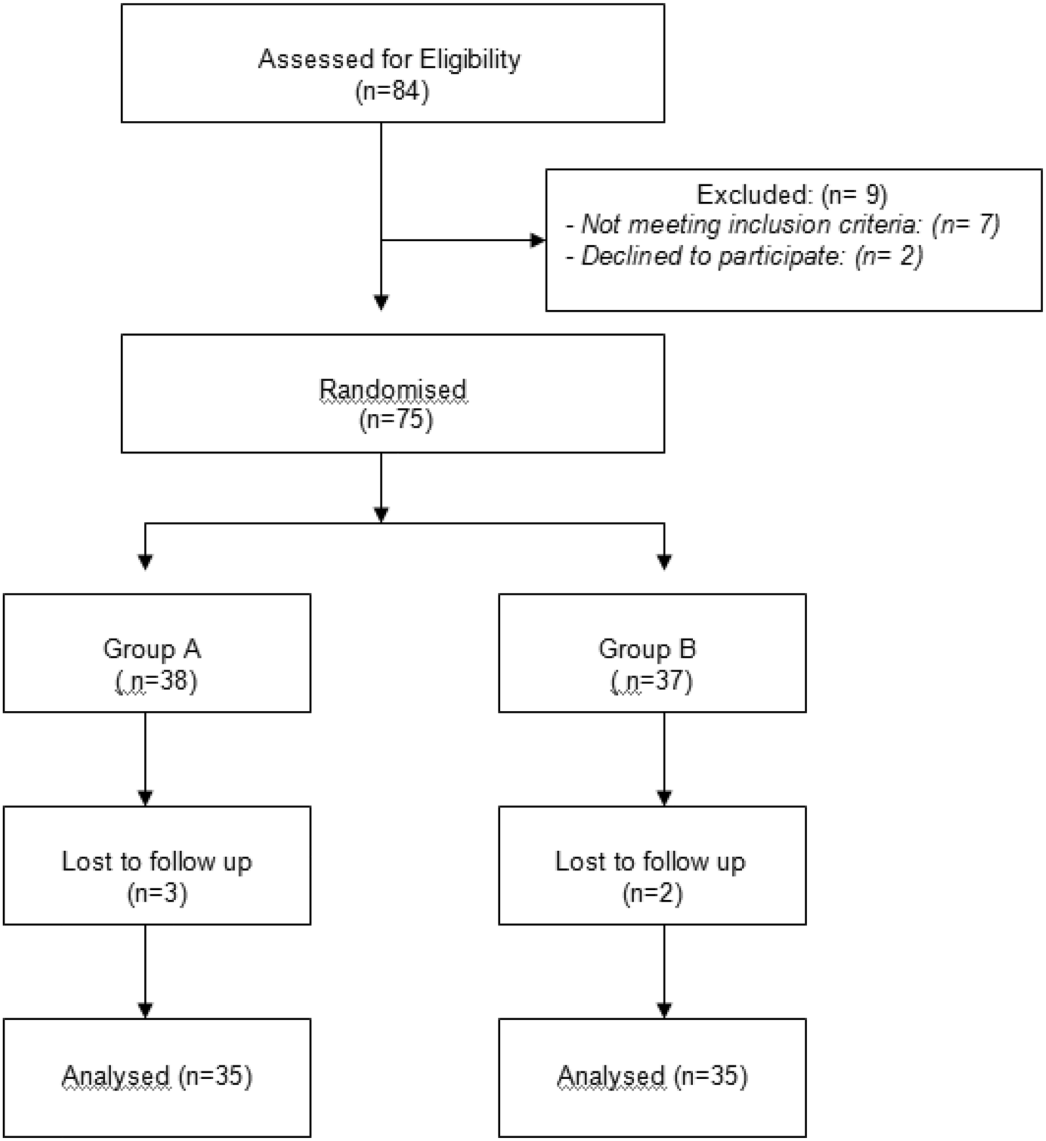
Flow of the patients through the study

Among 84 children tested for eligibility for enrollment in the study, nine patients were excluded for not meeting inclusion criteria or declining to participate in the study. A total of 75 patients were randomized to group A (38 patients) and group B (37 patients). Three patients in group A, and two in group B were lost to follow-up and were excluded from the study. The final analysis included 70 patients (35 in each group). Figure 1 shows the flow of the patients through the study.

There were no differences in demographic characteristics, operative duration, total fluoroscopy time and postoperative hospital stay between the two groups (Tables 1, 2).

**Table 1:** Demographic characteristics in both groups

	Group A (*n* = 35)	Group B (*n* = 35)	*P*-value
Age (years)	8.5 ± 1.5	9.3 ± 4.2	0.094
Sex, M/F	21/14	19/16	0.352
Stone site, R/L	20/15	18/17	0.182
BMI (kg/m^2^)	24.4 ± 1.8	25.0 ± 6.2	0.422
No. of stones, mean (SD),	1.4 (0.7)	1.7 (1.1)	0.262
Stone burden (mm)	23.2 ± 1.4	20.8 ± 3.5	0.579
Preop hemoglobin level (g/dL)	11.8 ± 0.2	11.4 ± 1.3	0.273

**Table 2:** Comparison of operative and postoperative events and outcomes in the two groups

	Group A (*n* = 35)	Group B (*n* = 35)	*P* value
Access time (min)	3.4 ± 1.2	1.4 ± 0.5	0.034*
Dilation fluoroscopy time (sec)	23.3 ± 8.4	8.8 ± 2.6	0.042*
Total fluoroscopy time (sec)	58.2 ± 3.5	41.6 ± 2.8	0.068
Failure of access	0	0	1
Operative duration (min)	67.4 ± 3.5 (38–102)	53.9 ± 4.6 (43–88)	0.785
Complications rate	10 (28.5%)	5 (14.2%)	
Intraoperative	5 (14. 2%)	1 (2.8%)	0.018*
Postoperative	5 (14. 2%)	4 (11.4%)	
Modified Clavien–Dindo grade			
Grade I			
Hematuria	2 (5.7%)	1 (2.8%)	
Grade II			
Bleeding requiring blood transfusion	6 (17.1%)	3 (8.5%)	
Urinary tract infection			
Grade III b			
Perforation	2 (5.7%)	1 (2.8%)	
Prolonged leakage			
Blood transfusion	4 (11.4%)	1 (2.8%)	0.045*
Stone free rate	33 (94.3%)	33 (94.3%)	1
Postoperative hospital stay (days)	1.4 ± 0.2	1.2 ± 0.6	0.182
Postop. hemoglobin level (g/dL)	10.8 ± 0.4	11.8 ± 1.1	0.613
Hemoglobin deficit (g/dL)	1.5 ± 0.5	0.6 ± 0.2	0.026*

*Significant

Table 2 summarizes operative and perioperative events and outcomes in both groups. Access was successfully obtained in all procedures. We did not encounter any difficulties in tract dilatation in both techniques. All the procedures were performed through a single tract. Access time and dilatation fluoroscopy time were shorter in group B than in group A. (statistically significant, *p* = 0.034 and 0.042, respectively).

The overall complication rate was 21.4% in all patients (28.5% in group A and 14.2% in group B), this was statistically significant (*p* = 0.018). Intraoperative complications included five patients with intraoperative bleeding requiring blood transfusion (four in group A (11.4%) and one (2.8%) in group B). The overall need for blood transfusion was significantly higher in group 1 (11.4 v 2.8%; *P* = 0.495). Perforation of the renal pelvis with urinary extravasation was observed in another patient in group A, necessitating double J stent placement and procedure termination. No organ injury was recorded in either group.

Postoperative complications included urinary tract infections in two patients (5.7%) in each group which was managed conservatively with appropriate antibiotics. Gross hematuria in two patients (5.7%) in group A and one (2.8%) in group B, which was managed by bed rest, intravenous fluids, and nephrostomy tube clamping.

Prolonged leakage of urine after removal of the nephrostomy tubes in one patient in each group which was managed with endoscopic double J stent insertion in one patient, and ureteroscopy and stone fragment disintegration and retrieval in another child. There were no major complications (modified Clavien IV or higher) among patients in both groups.

The mean hemoglobin deficit before and after surgery was significantly higher in group A (*p* = 0.026) (Table 2). Stone analysis was available for 32 procedures, and revealed calcium oxalate in 19 (59.3%), uric acid in 7 (21.8%), struvite in 4 (12.5%), and cystine in 2 (6.3%).

There overall stone-free rates at one month were similar for the two groups (33/35, 94.3%). Secondary procedures in the form of ESWL were done for two patients in group A, and one patient in group B.

## Discussion

The ideal technique for percutaneous tract dilatation during PCNL remains controversial. Endourologist may prefer and resort to a particular technique, depending on their familiarity or expertise. Four standard dilatation techniques are currently in use: metal telescopic dilators, Amplatz fascial dilators, balloon dilators and single-step technique [9–11].

Metal telescopic dilators are reusable and thus more economical. They maintain a tamponade effect throughout the dilation steps [12]. Amplatz fascial dilators are disposable, and it has been proposed that during sequential dilators exchange, the tamponade effect on the renal parenchymal is lost, which can lead to more intraoperative bleeding [3, 13]. However, no difference between Alken metal and sequential Amplatz fascial dilatation in terms of safety and efficacy has been reported [10, 11].

Both sequential metal and Amplatz fascial dilators have the disadvantages of prolonged access time associated with multiple incremental dilatation and more fluoroscopy exposure time. Also, more chances of tract displacement or loss due to kinking of the guidewire during axial force transmission while developing the tract [9–11].

Balloon dilatation is considered as the least time consuming and safest modality for dilatation [14]. It has many advantages as it is associated with less incidence of intraoperative bleeding and prevents renal displacement throughout tract creation. However, its high cost and being disposable limits its routine use. Failure rate of 17% was reported, and it increases to 25% in patient in whom there was previous renal surgeries [15].

To optimize percutaneous tract dilatation with reducing dilatation time and fluoroscopy exposure during the access, a single step technique was introduced. Multiple studies in adult patients have reported the safety and efficacy and less X-ray exposure time associated with the use of single step dilatation [12, 13, 16]. Multiple randomized controlled trials (RCTs) and two recent meta-analyses have demonstrated that the single step technique is as safe and effective as other techniques but is more cost effective and less time consuming [10–13, 17–19]. Significant differences were reported in fluoroscopy exposure time and access time between metal telescopic dilatation and single step dilatation.

On the contrary, several reports showed several unsuccessful attempts of single step dilatation in patients with history of previous renal surgery, due to high resistance encountered by dense perirenal fibrosis, which hindered fascial dilator passage [4, 11, 12]. Moreover, Aminsharifi et al. [14] showed that single step dilatation may have a substantial risk of serious complications including parenchymal damage and intraoperative bleeding compared to serial Alken metal dilatation especially with lack of endoscopic expertise [7].

In the present study, we encountered no difficulty in tract establishment and dilatation in both groups. All our patients had no history of previous renal surgery. A single surgeon with technical endoscopic experience performed all these procedures. This may explain favorable outcomes of single step dilation with no significant complications.

There are few publications in the literature addressing the use of single step dilatation in pediatric PCNL. Hosseini et al. evaluated the safety and efficacy of one-shot tract dilatation in preschool children. They compared fluoroscopy time, tract creation, dilatation time, success rate and complications between single step dilatation and serial metal dilatation. No significant differences were found regarding successful dilatation rate, access time, operative time, stone free ate and complications between the two groups. They concluded that one-stage dilatation technique is safe, effective and was associated with considerably less radiation exposure in preschool children [20].

In this study we compared a single-step dilatation technique which consists of a single dilatation of the track with a 20F dilator with serial metal Alken dilatation in children who underwent PCNL procedures. We used tract dilatation fluoroscopy time instead of total fluoroscopy time because the latter is affected by multiple factors including time needed for collecting system access, guidewires insertion, and the residual fragments retrieval and confirmation of complete stone clearance.

Our results were consistent with the above-mentioned study; as they clearly show the safety and efficacy of one-shot dilatation technique; the operative time, stone-free rate, and hospital stay were comparable in both groups. In the present study, we found that both access time and dilatation fluoroscopy time were significantly lower in patients from group B. Although the total fluoroscopy time was shorter in the group B, this difference was not statistically significant. These findings correlate with the results of RCTs addressing single step dilation technique in adults [17–19].

The idea of optimization fluoroscopy exposure during diagnostic and interventional endourological procedures has recently been highlighted, especially in vulnerable population like children [21, 22]. The potential hazards associated with repeated and prolonged radiation exposure is substantially increased in the pediatric population. Children are more radiosensitive than adults and have more life expectancy, during which they might experience radiation-related complications [23, 24]. Therefore, any protective measures aiming at minimizing those hazards should be employed and adopted.

One of the major complications encountered during PCNL is intraoperative bleeding, which ranges from mild to severe hemorrhage requiring blood transfusion. Many variables may impact the incidence of PCNL-related intraoperative bleeding including tract size, number of the tracts, stone burden, operative time, and surgeon expertise [5–7]. Nour et al. reported in his study comparable rates of bleeding and other complications between single‑step renal dilatation and serial dilatation [17]. Single-step and stepwise tract dilatation were compared in terms of the impact of the chosen technique on intraoperative bleeding among two groups of patients [18]. Although hemorrhage necessitating blood transfusion rates were lower in the single-step dilatation group, the difference did not reach statistical significance.

The rate of transfusions during PCNL varies among different series. No hemorrhagic complications requiring transfusion was reported by Fraser et al. [25]. In contrast, Özden et al. reported 24% need for blood transfusion in the first 25 patients, which decreased to 10% in the following 28 patients during PCNL in children with complex renal stones [26]. In the present study, patients in group A had a significantly higher rates of complications compared to patients in group B (28.5 versus 14.2%). Intraoperative bleeding requiring blood transfusion was statistically higher in group A compared to group B (11.4 versus 2.8%, *P* = 0.045). This may be attributed to less chance of tissue trauma induced by single step dilatation.

We noticed that tract dilatation usually stops short to the pelvi-calyceal system, with no incidence of injury or perforation as might be induced by serial metal dilators especially in the hand of experienced endourologist. One patient in group A developed perforation of the renal pelvis during the dilatation requiring procedure termination and insertion of a double J stent. This was probably due to the tract overdilation over the working guidewire. This patient needed two sessions of ESWL to render him stone free. The main postoperative complications included urinary tract infections, hematuria, and prolonged urine leakage in the current study.

The present study may be limited by the absence of a cost-effective analysis comparison for the two dilatation methods. Of note, only a single disposable dilator is needed to establish a tract with the single step technique, however, Alken metal dilators are reusable and therefore more economic. A variety of factor, including the stone burden, body mass index, hydronephrosis grade and the operative duration may have influenced the incidence of intraoperative bleeding and subsequent blood transfusion; subgroup analyses were not performed due to relatively few numbers of patients. However, one of the strengths of the current study is that all cases were performed by a single surgeon who had an expertise in pediatric PCNL. The third limitation is the single-center experience with short-term follow up time.

Our results showed that the single-step dilatation method can reduce pediatric patients’ exposure during the access phase to radiation without affecting the safety or the efficacy of dilation process. Moreover, this was associated with significantly lower overall complication rate, notably, intraoperative bleeding requiring blood transfusion, rendering this technique a safer and better option for tract dilatation during pediatric PCNL. The efficacy and reliability of this technique in children should be verified with larger future prospective studies and with long-term follow up.

## Conclusions

Compared with serial metal track dilatation, single step dilatation technique showed comparable operative duration and stone free rate during pediatric PCNL, with significantly reduced access time and dilatation fluoroscopy time. Intraoperative bleeding requiring blood transfusion was significantly lower in single step dilatation group.
